# Effects of kaempherol-3-rhamnoside on metabolic enzymes and AMPK in the liver tissue of STZ-induced diabetes in mice

**DOI:** 10.1038/s41598-024-66426-x

**Published:** 2024-07-13

**Authors:** Alhussain H. Aodah, Faisal K. Alkholifi, Khalid M. Alharthy, Sushma Devi, Ahmed I. Foudah, Hasan S. Yusufoglu, Aftab Alam

**Affiliations:** 1 Department of Pharmaceutics, College of Pharmacy, Prince Sattam Bin Abdulaziz University, 11942 Al Kharj, Saudi Arabia; 2 Department of Pharmacology and Toxicology, College of Pharmacy, Prince Sattam Bin Abdulaziz University, 11942 Al Kharj, Saudi Arabia; 3 Chitkara College of Pharmacy, Chitkara University, Rajpura, Punjab 140401 India; 4 Department of Pharmacognosy, College of Pharmacy, Prince Sattam Bin Abdulaziz University, 11942 Al Kharj, Saudi Arabia; 5 Department of Pharmacognosy and Pharmaceutical Chemistry, College of Dentistry and Pharmacy, Buraydah Private Colleges, 51418 Buraydah, Saudi Arabia

**Keywords:** Kaempherol-3-rhamnoside, Diabetes, Glycolytic enzymes, AMPK, Insulin, Biochemistry, Molecular biology, Endocrinology

## Abstract

Diabetes mellitus (DM) is a chronic metabolic disorder characterized by persistent hyperglycemia. It involves disturbances in carbohydrate, fat, and protein metabolism due to defects in insulin secretion, insulin action, or both. Novel therapeutic approaches are continuously being explored to enhance metabolic control and prevent complications associated with the disease. This study investigates the therapeutic potential of kaempherol-3-rhamnoside, a flavonoid, in managing diabetes by modulating the AMP-activated protein kinase (AMPK) pathway and improving metabolic enzyme activities in streptozotocin (STZ) -induced diabetic mice. Diabetic mice were treated with varying doses of kaempherol-3-rhamnoside and/or insulin over a 28-day period. Glycolytic and gluconeogenesis enzyme activities in the liver, fasting blood glucose levels, serum insulin levels, lipid profiles and oxidative stress markers were assessed. Treatment with kaempherol-3-rhamnoside significantly improved glycolytic enzyme activities, reduced fasting blood glucose, and enhanced insulin levels compared to diabetic controls. The compound also normalized lipid profiles and reduced oxidative stress in the liver, suggesting its potential in reversing diabetic dyslipidemia and oxidative damage. Furthermore, kaempherol-3-rhamnoside activated the AMPK pathway, indicating a mechanism through which it could exert its effects. Kaempherol-3-rhamnoside exhibits promising antidiabetic properties, potentially through AMPK pathway activation and metabolic enzyme modulation. These findings support its potential use as an adjunct therapy for diabetes management. Further clinical studies are warranted to validate these results in human subjects.

## Introduction

Diabetes mellitus (DM) refers to a cluster of metabolic disorders that cause elevated levels of glucose, lipids, amino acids, and lactate circulating in the body. The root causes of DM include inadequate insulin secretion from the pancreas or diminished sensitivity to insulin or both simultaneously. It affects both insulin secretion and action resulting in hyperinsulinemia^1,2^. According to the World Health Organisation (WHO), it is predicted that by 2030, there will be more than twice as many people worldwide who have DM compared to the numbers recorded in 2000. The estimated figure could surpass a staggering total of 300 million people affected by this condition^3^.

To effectively manage diabetes, the ultimate aim is to regulate blood sugar levels in both type 1 and type 2 cases^4^. Achieving this requires efficient glycolysis, which transforms glucose into energy that manages insulin and cell activity. Enzymes such as glucokinase and 6-phosphofructo-1-kinase control the glycolysis rate. Enzymes vary in their regulation between cell types. The 6-phosphofructo-2-kinase/fructose-2,6-bisphosphatase enzyme is also crucial in this process^5^. Enzymes respond to signals from nutrition and hormones, which affect their transcription, translation, and post-translational modifications. The intricate process of hepatic glucose production is essentially a result of the complex interplay between enzymes and metabolic pathways within hepatocytes, which work together to regulate glycolysis^6^. When there is too much, the latter can lead to high blood sugar levels in people with diabetes. Glycolysis results in glucose-stimulated insulin release in pancreatic beta cells. Hyperglycemia can occur when there is a deficiency of circulating insulin or when its function is impaired^5^. Adipocytes use glycolysis to produce metabolites that facilitate lipogenesis and redirect surplus fatty acids toward the synthesis of triglycerides. This mechanism curtails oxidative stress within cells. Adipocytes generate pro-hyperglycemic factors when the body is in a state of increased inflammation, leading to insulin resistance and elevated blood glucose levels^7^.

When insulin levels are inadequate or the body becomes resistant to it, there is a malfunctioning of glycolysis. This is caused by the metabolic and regulatory enzymes involved in glycolysis functioning inappropriately in terms of quantity and/or activity. Effective strategies to manage diabetes and its associated complications may include measures to improve glycolytic activity by modulating key metabolic and regulatory enzymes^8,9^. The association of enzymes affects glucose oxidation. The high association of enzymes favours glucose for fermentation and biosynthesis. In skeletal and cardiac muscles of diabetic patients, glycolysis undergoes down-regulation due to hexokinase and phosphofructokinase enzyme deficiency. Cancer conditions commonly show elevated glycolytic rates associated with this phenomenon^6^.

The appropriate utilization of glucose by various tissues in the body, including the liver, pancreas, and kidneys, is critical in diabetes to prevent the accumulation and toxicity of cellular glucose of cellular glucose that can lead to serious complications. This highlights the importance of regulating both under- and over-use of glucose. Insulin-dependent diabetes mellitus is characterised by metabolic dysfunction primarily resulting from hyperinsulinemia and hyperglycemia. The maintenance of carbohydrate and lipid homeostasis depends on the equilibrium between their production and usage in the main peripheral tissues, which is substantially altered in individuals with diabetes^10–12^. This mechanism is key to developing new antidiabetic drugs. The 5′-adenosine monophosphate-activated protein kinase (AMPK) signaling pathway effectively regulates diabetes. Activation of the AMPK protein can increase glucose uptake and suppress intracellular glucose production. This promotes optimal cell function, as shown in several studies. Impaired AMPK activity is common among people with diabetes, making activation of this protein a persuasive solution. In particular, drugs used to treat this condition, including metformin, exert their therapeutic effects by regulating AMPK function. As a result, pharmacological substances with promising potential as therapeutic choices for the treatment of DM include those that can efficiently activate and modulate AMPK activity^13,14^. It is important to consider alternative treatments based on scientific research for patients who experience adverse effects from allopathic drugs, including hypoglycemia. Plant-based medications such as berberine, quercetin, and resveratrol can effectively regulate the AMPK pathway to manage diabetes mellitus without harmful side effects^15^.

**Figure 1:**
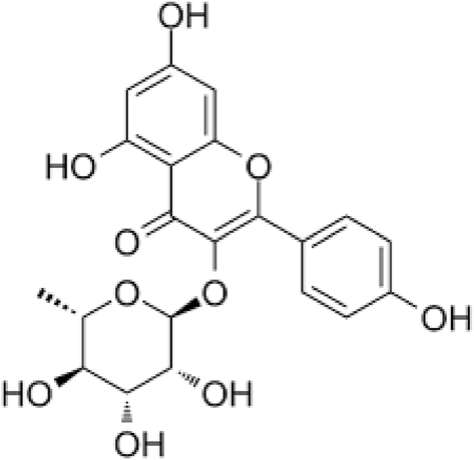
The chemical structure of kaempferol-3-rhamnoside.

Insulin therapy and oral drugs such sulfonylureas, biguanides, -glucosidase inhibitors, and glinides are often used to manage diabetes. However, in resource-limited settings where access to these drugs is limited due to high costs and unavailability; adopting alternative therapies or preventive measures becomes crucial. To provide optimal diabetes care, healthcare providers should explore lifestyle changes and natural remedies^16^. In the world of healthcare, there is a new wave of interest in herbal remedies. This can be attributed to the harmful side effects associated with taking oral hypoglycemic agents, which are often prescribed to treat diabetes mellitus. With more and more people looking for natural alternatives to traditional medications, it is crucial that we embrace this shift towards holistic healing methods. Herbal remedies have been used for centuries to treat various ailments and now offer a safe solution for those seeking relief from their diabetic symptoms without risking dangerous side effects. Traditional plant-based remedies are vital in treating DM. Based on scientific evidence, these traditional remedies have proven effective in controlling blood sugar levels and alleviating the harmful effects of this persistent medical condition^17,18^. Kaempferol-3-rhamnoside is a flavonoid glycoside that belongs to the class of phytochemicals known as flavonoids, recognized for their medicinal properties and health benefits. It is derived primarily from the sources of *Ficus palmata* and *Nymphaea odorata*^19^. The molecule kaempferol-3-rhamnoside is a flavonoid glycoside comprised of the flavonol kaempferol attached to a rhamnose sugar molecule^20^. The kaempferol core features three hydroxyl groups linked to its two benzene rings and a heterocyclic ring. This compound is glycosylated at the 3-position where the rhamnose is bonded through an oxygen atom^21^. The functional groups, particularly the hydroxyl groups, are key to the molecule's solubility, reactivity, and overall biological activity. The chemical structure of kaempferol-3-rhamnoside is given in Fig. 1.

Kaempherol-3-rhamnoside is an exceptional natural compound that acts as both a potent inhibitor of NOS and NADPH oxidase. Its impressive therapeutic potential is attributed to its remarkable multifunctional properties, which include powerful antibacterial, anti-inflammatory, antiapoptotic, and antitumour activities. It is no wonder why kaempherol-3-rhamnoside continues to be a promising candidate for future drug development efforts. kaempherol-3-rhamnoside might serve as an effective treatment against *P. aeruginosa*-related diseases^22^. While promising results have been observed in animal studies, further research is needed to determine the efficacy and safety of kaempherol-3-rhamnoside as a potential treatment for breast^23^. We investigated the effects of kaempherol-3-rhamnoside on metabolic and oxidative enzymes in diabetic mice. This study is significant to understanding the benefits of using kaempherol-3-rhamnoside to manage diabetes, which could lead to more effective treatments.

## Materials and methods

### Chemicals

Streptozotocin purchased from Sisco Research Laboratories Pvt. Ltd., India. Glucose kit (120200- Erba Mannheim, India), Insulin ELISA Kit (Millipore, Billerica, MA, USA) were used. Cholesterol, triglycerides, high-density lipoproteins and free fatty acids were assessed using assay kits purchased from Spin react, Spain. Kaempherol-3-rhamnoside was obtained as gift sample from Humed life sciences. Anti-AMPK and anti-p-AMPK from Abcam Inc., Cambridge, MA. The enzyme kits were purchased from: hexokinase type I enzyme—ab136957, Colorimetric assay kit from Abcam Inc.); phosphofructokinase- MAK093, and pyruvate kinase—MAK072 from Sigma Aldrich, India; lactate dehydrogenase, glucose-6-phosphatase and fructose-1,6-bisphosphatase from BT LAB, Bioassay Technology Laboratory, India. It is imperative that all reagents administered to animals must adhere to pharmacological and chemical grade standards, ensuring they are free from contamination.

### Acute oral toxicity study

A study was conducted to evaluate the acute oral toxicity of the substance in accordance with guidelines provided by the OECD (Organisation for Economic Cooperation and Development) No. 423. In this study, albino mice with weight ranging from 18 to 28 g and male gender were used. To ensure consistency in drug administration and minimize variability between individuals, all mice underwent an overnight fast prior to receiving any drugs or treatments. Three mice received a robust single oral dose equivalent to 500 mg/kg of their body weight, courtesy of the powerful compound known as kaempherol-3-rhamnoside. After giving kaempherol-3-rhamnoside, food was not allowed for the next 4–5 h. The animals were carefully monitored as individuals within the first half hour of being dosed and then at various intervals throughout the initial 24 h (with a particular focus on the first four). Following this initial period, they were observed daily for 2 weeks. Overall, their well-being was closely monitored over an extended period of time. Daily, observations were made on the animal’s appearance and behaviour including skin, fur, eyes, nose, breathing rate, heart rate, piloerection (hair standing), involuntary urination or defecation along with changes in drowsiness, gait, tremors, and seizures. Any deaths that occurred within 14 days were recorded^24^.

### Animals

This study was conducted using male albino mice, weighing 18–28 g and aged 7–8 weeks. The mice were housed in a temperature-controlled environment (22 ± 2 °C) with a 12-h light–dark cycle. Ad libitum access to commercially available rodent food from the local market of Saudi Arabia and tap water was provided. Food were replenished daily to ensure constant availability. All methods and animal care procedures were carried out in strict accordance with the ARRIVE guidelines (https://arriveguidelines.org). Additionally, this protocol was approved by the Internal Animal Ethics Committee under authorization number SCBR-24/2022, ensuring all ethical and welfare considerations were met.

### Experimental design

#### Induction of diabetes

The development of DM was begun by administering a single ip dose of 65 mg/kg STZ that had been dissolved in citrate buffer (100 mM, pH 4.5) according to the meth-od described in the reference source provided^25^. All animals involved in the experiment were fed a high-fat diet, except the normal control group, who received citrate buffer only. After four days, mice that showed hyperglycemia (measured at ≥ 250 mg/dL) were classified as diabetic. The animals were divided into the following groups:

- Group I: Normal control, received vehicle only.
- Group II: Diabetic control, received streptozotocin (STZ) only.
- Group III: Diabetic mice treated with regular human insulin at 2 U/kg daily for 28 days.
- Group IV: Diabetic mice treated with kaempherol-3-rhamnoside at 2.5 mg/kg daily for 28 days.
- Group V: Diabetic mice treated with kaempherol-3-rhamnoside at 5 mg/kg daily for 28 days.
- Group VI: Diabetic mice treated with a combination of human insulin (1U/kg) and kaempherol-3-rhamnoside (2.5 mg/kg) daily for 28 days.

Mice underwent 28 days of treatment, after which they were fasted and weighed. Blood samples were then collected from the retroorbital area. Following anesthesia, the mice were euthanized by cervical decapitation^26,27^. Subsequently, the pancreas and liver tissue were isolated, weighed and stored in a deep freezer with a temperature of − 70 °C^28,29^.

### Fasting blood glucose level

The fasting blood glucose level was recorded on days 0, 14, 21 and 28. Blood samples were taken from the retro-orbital area, and the findings were reported as milli-grams per deciliter (mg/dl)^30^. Blood glucose levels were measured using a glucometer (Accu-Chek).

### Plasma insulin level

On the 28th day, plasma insulin levels were measured using a Rat/Mouse Insulin ELISA Kit (Millipore, Billerica, MA, USA) and reported as µIU/mL^30^.

### Preparation of tissue extracts for enzyme assays

To prepare tissue extracts, animals from each group were starved overnight and then euthanized. The liver was washed in saline, weighed, minced, and homogenized in a cold isotonic sucrose buffer using a tissue homogenizer. The homogenization buffer contained 0.25 M sucrose, 0.02 M triethanolamine, and 0.12 mM dithiothreitol to enhance uniformity, with a pH of 7.4. All procedures were performed at 4 °C. The homogenate was then centrifuged for 10 min at 1000 g. The supernatant from this centrifugation was further centrifuged at 105,000*g* for 45 min at 4 °C. The clear fraction of the supernatant was collected for enzyme assays^31^.

### Glycolytic/metabolic enzyme assays in liver

Coupled enzymatic reaction systems were utilised to estimate the activity of hexokinase isozymes in the supernatant fraction by spectrophotometric measurement, according to the Gumaa and McLean method^32^. Phosphofructokinase^33^, pyruvate kinase and lactate dehydrogenase^34^ was carried out as per paper published earlier. The gluconeogenic enzymes glucose-6-phosphatase and fructose-1,6-bisphosphatase were tested using the methods of Baginsky et al.^35^ and Tashima and Yoshimura^36,37^, respectively. The NADP-linked lipogenic enzyme glucose6-phosphate dehydrogenase was essentially determined by the method of Baquer et al.^38^. An enzyme unit is equal to the formation of 1 µmole of NAD/NADH or NADP/NADPH per gram of fresh tissue weight each minute at a temperature of 25 °C. Similarly, for glucose-6-phosphatase and fructose-6-bisphosphatase, one unit refers to the liberation amount of Pi per gram fresh weight every minute while being subjected to a temperature equivalent to 37 °C.

### Lipid profile

Serum levels of cholesterol (CHO), triglycerides (TG), high-density lipoproteins (HDL) and free fatty acids (FFA) were accurately assessed using Spin react assay kits, Spain. Comprehensive testing helped to gain a complete understanding of the metabolic health of the participants. These evaluations provided significant information on the lipid profile of each participant, which can be used to target specific aspects of their risk factors. Each milligram per deciliter (mg/dl) measurement was meticulously examined to ensure data collection accuracy and precision. In addition, the advanced features integrated with the assay kit streamlined the processes by allowing seamless execution and reducing processing time^39^.

### Lipid peroxidation assay

The presence of malondialdehyde (MDA) in tissue was evaluated through the utilisation of a reactive substance of thiobarbituric acid (TBA) at an elevated temperature, producing a pigmented compound. The amount was quantified as nanomoles per milligram of tissue using a molar absorption coefficient set at 156,000 M^−1^ cm^−1^ with λ = 532 nm. The mixture included phosphate buffer, potassium permanganate (1 mM), and the sample. The pH was 7.4 at a concentration of 10 mM for the buffer. To start the reaction, ferrous sulphate (10 mM) was added twice. The reaction was stopped by adding trichloroacetic acid (20%). Malondialdehyde (MDA) reacted with TBA to produce a coloured product^40^.

### Estimation of TNF- α

ELISA kits were used to estimate the levels of TNF-α, which is an inflammatory cytokine found in tissues. Manufacturer instructions were followed during this process.

### Antioxidant enzymes assays

The evaluation of superoxide dismutase (SOD) was conducted using the technique described by Marklund and Marklund^41^, with results expressed in units per milligram of protein. Catalase activity (CAT) was measured following Sinha’s (1972) methodology^42^, and the results were presented as units per milligram of protein. Glutathione peroxidase (GPx) levels were assessed by measuring the activity, expressed as the amount of glutathione utilized per minute per milligram of protein^43^.

### Total AMP-activated protein kinase (AMPK) and phospho-AMPK (p-AMPK) proteins

A liver supernatant homogenate, which contained 20 mg of protein, was subjected to SDS-PAGE and then transferred to a nitrocellulose membrane by electrophoresis. The NC membrane was blocked for 2 h at room temperature before being incu-bated with polyclonal antibodies against anti-AMPK and anti-p-AMPK from Abcam Inc., Cambridge, MA, overnight at 4 °C. Following this process, the membranes were treated with horseradish-peroxidase-conjugated antirabbit IgG. Ultimately, Nitrocellulose membranes received a thorough wash in solution for half an hour, while immunoreactive lanes detected by Band Scan software version 5.0 were enhanced using chemiluminescence method before digitalization took place^44^.

### Statistical analysis of data

The results were presented as average ± standard error of the mean. A one-way analysis of variance was employed to establish the significance between two values, and statistical significance was defined at a level of *p* < 0.05. The evaluations were executed using Graphic Pad prism 7.0 software.

### Institutional Review Board Statement

All procedures regarding animal care and treatment conformed to the Animal Care Guidelines of the Standing Committee on Bioethics of Prince Sattam Bin Abdulaziz University (SCBR) 24/2022), Al-Kharj, Ministry of Education, Kingdom of Saudi Arabia.

## Results

### Acute toxicity study

Based on the experiments conducted, it appears that the administration of kaempherol-3-rhamnoside to animals did not result in any harmful symptoms or death observed. These findings suggest that the LD_50_ value for kaempherol-3-rhamnoside is likely above 200 mg/kg body weight, indicating its safety and suggesting the potential use as a compound for further research in pharmacology. To proceed with this study, 2.5 mg/kg of 2.5 mg/kg p.o. and 5 mg/kg of weight were chosen for additional investigation based on their relevance to expected physiological effects within an animal model system under experimental conditions.

### Effect of kaempherol-3-rhamnoside on fasting blood glucose (FBG) level

**Figure 2:**
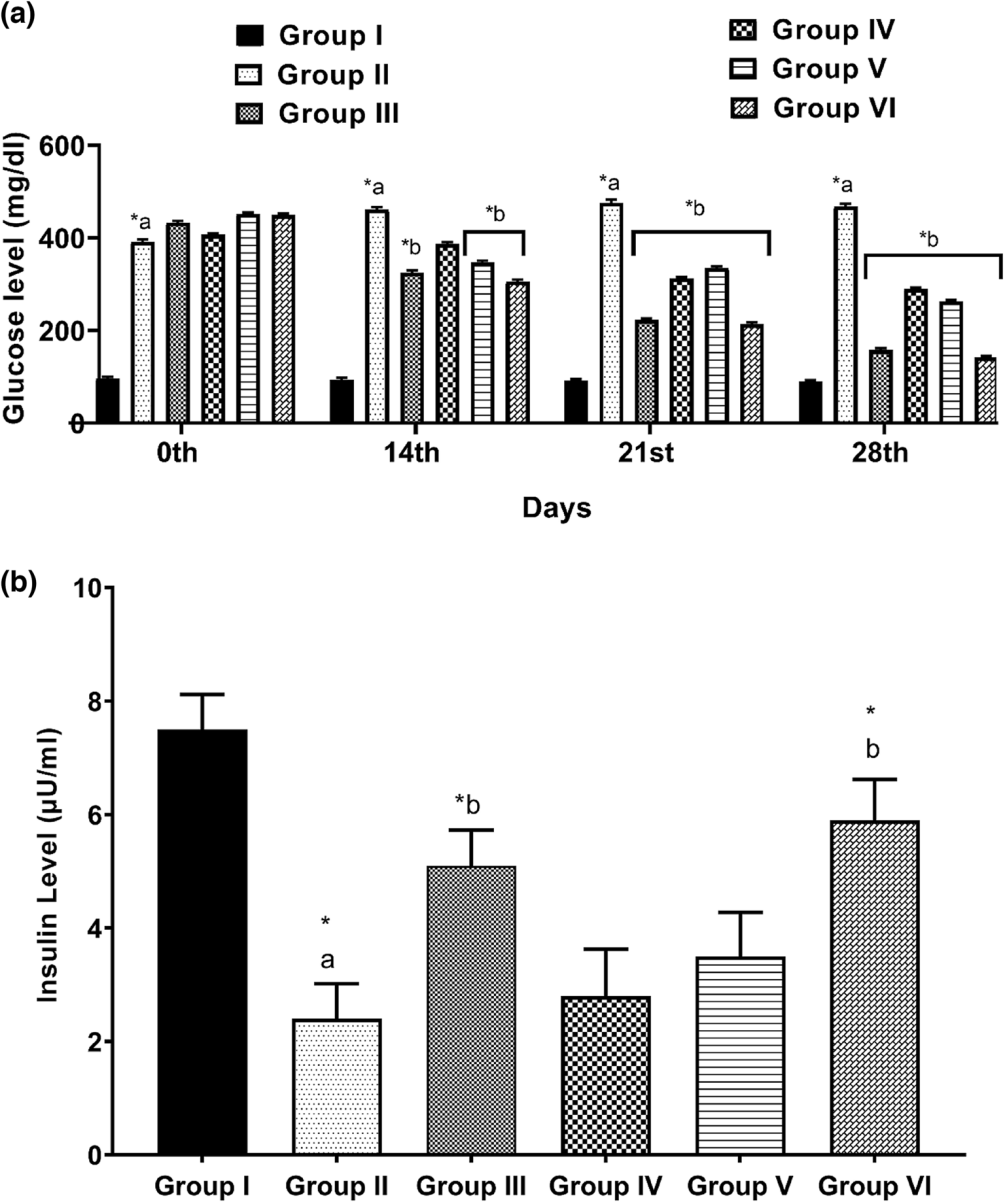
(**a**) Effect of kaempherol-3-rhamnoside and its combination on glucose level was analyzed. Statistical significance was determined by a *p*-value less than 0.05 (*p < 0.05). The following pairs were compared for statistical significance: a) normal control versus diabetic control corresponding to the same day, b) diabetic control versus treatment groups corresponding to the same day; (**b**) Effect of kaempherol-3-rhamnoside and its combination on insulin level was studied. The statistical significance threshold was set at a *p*-value of less than 0.05, denoted as *p < 0.05 in the results section. Comparison between treatment groups and diabetic control (b) as well as normal control (a) were analyzed for significant differences in insulin levels.

Figure 2a illustrates the impact of a 28-day study on FBG in various experimental groups. The presence of high fasting glucose levels confirmed diabetes induction in the mice, compared to the normal group. After treating diabetic mice with kaempherol-3-rhamnoside for four weeks, there was a significant reduction (*p* < 0.05) reduction in their blood sugar levels compared to those of the diabetic control group, indicating its effectiveness as a treatment option. Furthermore, the effect of kaempherol-3-rhamnoside appeared to be dose-dependent.

### Plasma insulin level

The results of the study examining the impact of kaempherol-3-rhamnoside and insulin on serum insulin levels in mice are shown in Fig. 2b. Before treatment, normal control mice had an average insulin level of 7.81 µU/mL. During the course of 28 days, diabetic mice experienced a notable decrease in serum insulin levels. However, administering kaempherol-3-rhamnoside orally daily resulted in serum insulin levels (3.89 µU/mL), but a combination of insulin + kaempherol-3-rhamnoside resulted in a higher insulin level (5.91 µIU/mL) as compared to untreated diabetic controls.

### Hepatic glycolytic/metabolic enzyme assays

**Figure 3:**
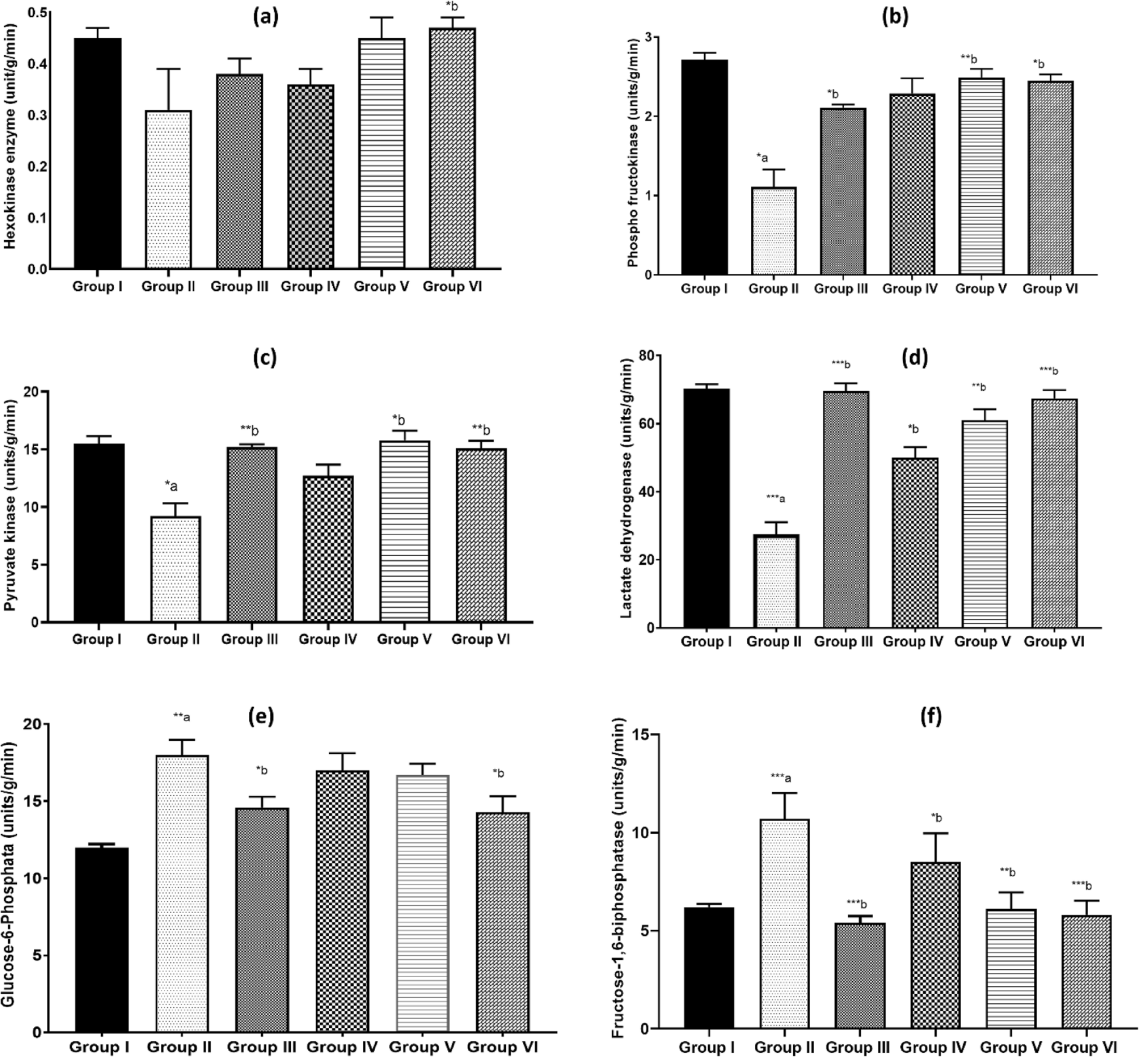
Effect of kaempherol-3-rhamnoside and its combination was studied on metabolic enzymes: (**a**) hexokinase type I enzyme; (**b**) phosphofructokinase; (**c**) pyruvate kinase; (**d**) lactate dehydrogenase; (**e**) glucose-6-phosphatase and; (**f**) fructose-1,6-bisphosphatase. Any *p*-value less than 0.05 was considered statistically significant (*p < 0.05; **p < 0.01; ***p < 0.001). Here a represents normal control versus diabetic control while b represents diabetic control versus treatment groups comparison without plagiarism involved in the content.

After being treated with kaempherol-3-rhamnoside and kaempherol-3-rhamnoside + Insulin for 28 days, diabetic mice showed metabolic activities similar to those of the normal control group. In diabetic mice, there was a significant decrease in overall levels of insulin-sensitive type I hexokinase enzyme present in their liver. Although not significantly different from that of normal controls, the level of hexokinase type I enzyme decreased among diabetic control groups. However, the treatment groups showed a statistically significant increase primarily when receiving combination therapy. These concise findings are illustrated in Fig. 3a.

Following 28 days of treatment, significant elevations were observed in the activity levels of three key enzymes involved in glucose metabolism-phosphofructokinase, pyruvate kinase, and lactate dehydrogenase-in the livers of diabetic mice treated with kaempferol-3-rhamnoside, compared to untreated diabetic controls. This enhancement in enzymatic activity was statistically substantiated by a *p*-value of less than 0.001 (*p* < 0.001), as illustrated in Fig. 3b–d. Diabetic mice administered either kaempferol-3-rhamnoside alone or in combination with insulin exhibited enzyme activities comparable to those observed in the nondiabetic control group. Collectively, these findings indicate that kaempferol-3-rhamnoside may serve as a promising therapeutic agent for addressing diabetes-associated metabolic dysfunctions in the liver.

In diabetic mice, there was a significant increase in the activity of glucose-6-phosphatase and fructose-1,6-bisphosphatase, enzymes critical for gluconeogenesis, within the cytosolic fractions of their livers^45^, evidenced by a *p*-value of less than 0.001. Treatment with kaempferol-3-rhamnoside alone or in combination with insulin for 28 days normalized the activities of these gluconeogenic enzymes as well as those of NADH-dependent lipogenic enzymes to levels observed in the livers of non-diabetic control mice, as demonstrated in Fig. 3e and f.

### Lipid profile

**Figure 4:**
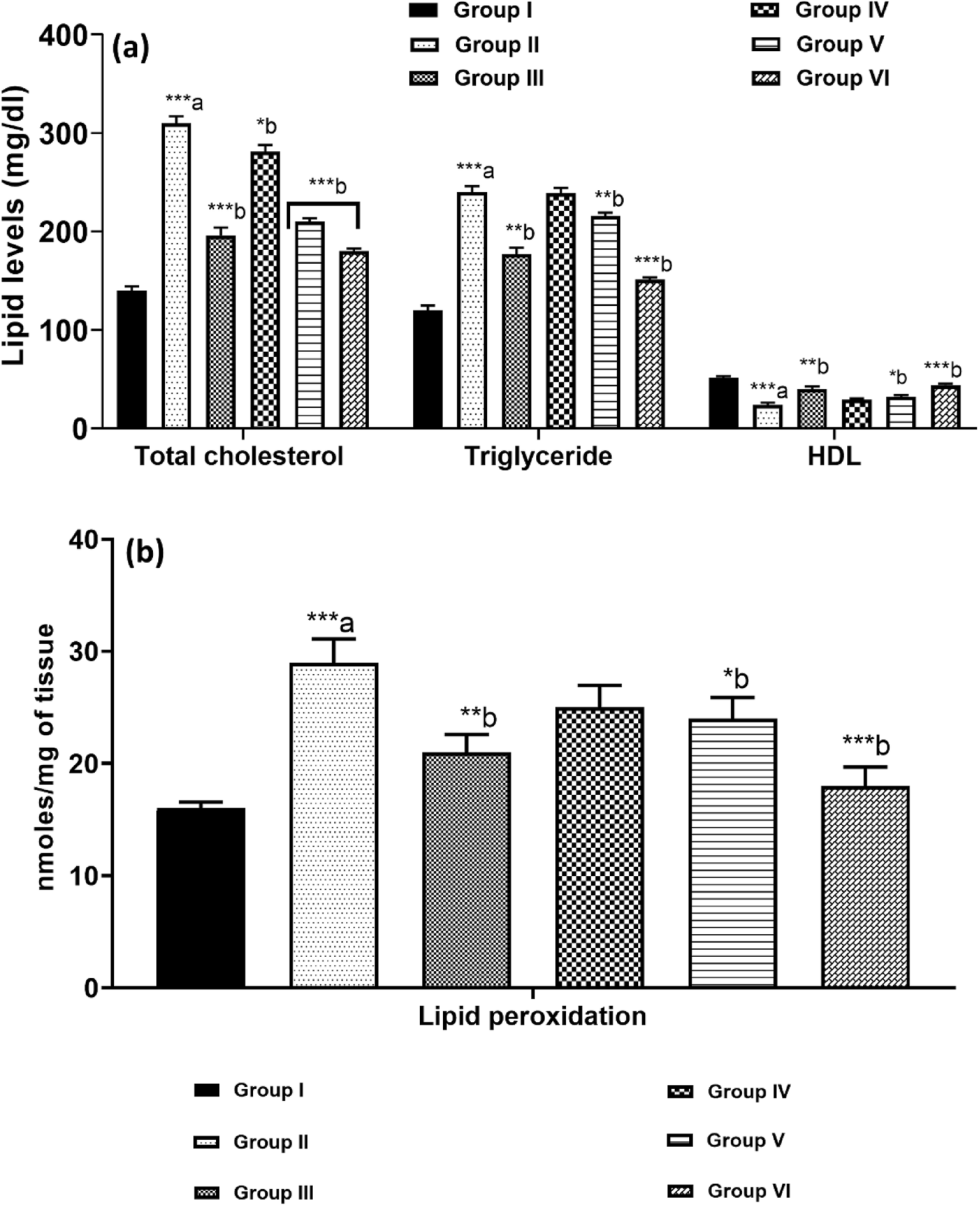
(**a**) Effect of kaempherol-3-rhamnoside and its combination on lipid levels was investigated, and statistical significance was determined using a *p*-value threshold of 0.05 (*p < 0.05; **p < 0.01; ***p < 0.001). The normal control group was compared to the diabetic control group (a), as well as to the treatment groups; (**b**) The impact of kaempherol-3-rhamnoside and its amalgamation on lipid peroxidation was analyzed, considering a *p*-value less than 0.05 as statistically significant. The significance values are expressed as *p < 0.05; **p < 0.01; ***p < 0.001 for the comparison between a-normal control versus diabetic control and b-diabetic control versus treatment groups.

The results shown in Fig. 4a indicate that the serum lipid profiles of the diabetic control group experienced significant pathological changes after the experimental period. Compared to the control groups, this group displayed significantly higher levels of total cholesterol and triglycerides (*p* < 0.001), along with markedly lower levels of HDL, which are key biomarkers for diabetes. These findings highlight the urgent need for interventions to improve the serum lipid profiles in the diabetic control group to enhance health outcomes. The study conclusively demonstrated that treatment with kaempferol-3-rhamnoside, both as a monotherapy and in conjunction with insulin, significantly attenuated elevated serum lipid concentrations, specifically total cholesterol and triglycerides, with a statistical significance of (*p* < 0.001). Administration of kaempferol-3-rhamnoside at a lower dosage of 2.5 mg/kg resulted in only a marginal improvement in total cholesterol levels (*p* < 0.05). However, the combination of kaempferol-3-rhamnoside with insulin was markedly more effective, leading to substantial reductions in both total cholesterol and triglyceride levels, and significantly enhancing serum HDL concentrations (*p* < 0.01) and (*p* < 0.001), respectively). These results indicate that the combined use of kaempferol-3-rhamnoside and insulin is superior to their individual applications for the management of hyperlipidemia.

### Lipid peroxidation in pancreas

Diabetes is characterized by enhanced lipid peroxidation, contributing to tissue damage and playing a role in the pathogenesis of both Type I and Type II diabetes^46^. Our study demonstrated elevated lipid peroxidation levels in the diabetic control group. However, treatment with kaempferol-3-rhamnoside and insulin significantly mitigated these levels, restoring them to near-normal, as illustrated in Fig. 4b. While low concentrations of lipid peroxides might stimulate insulin secretion, excessive levels can induce uncontrolled lipid peroxidation, leading to cellular damage within the pancreas. This damage demonstrates as complications associated with diabetes, including cellular infiltration and destruction of islet cells. The effects of kaempferol-3-rhamnoside and its combinations on lipid peroxidation were assessed, with statistical significance set at a *p*-value less than 0.05. Significance levels are denoted as *p < 0.05, **p < 0.01, and ***p < 0.001 for comparisons: (a) between normal control and diabetic control groups, and (b) between diabetic control and treatment groups.

### Antioxidant parameters

**Figure 5:**
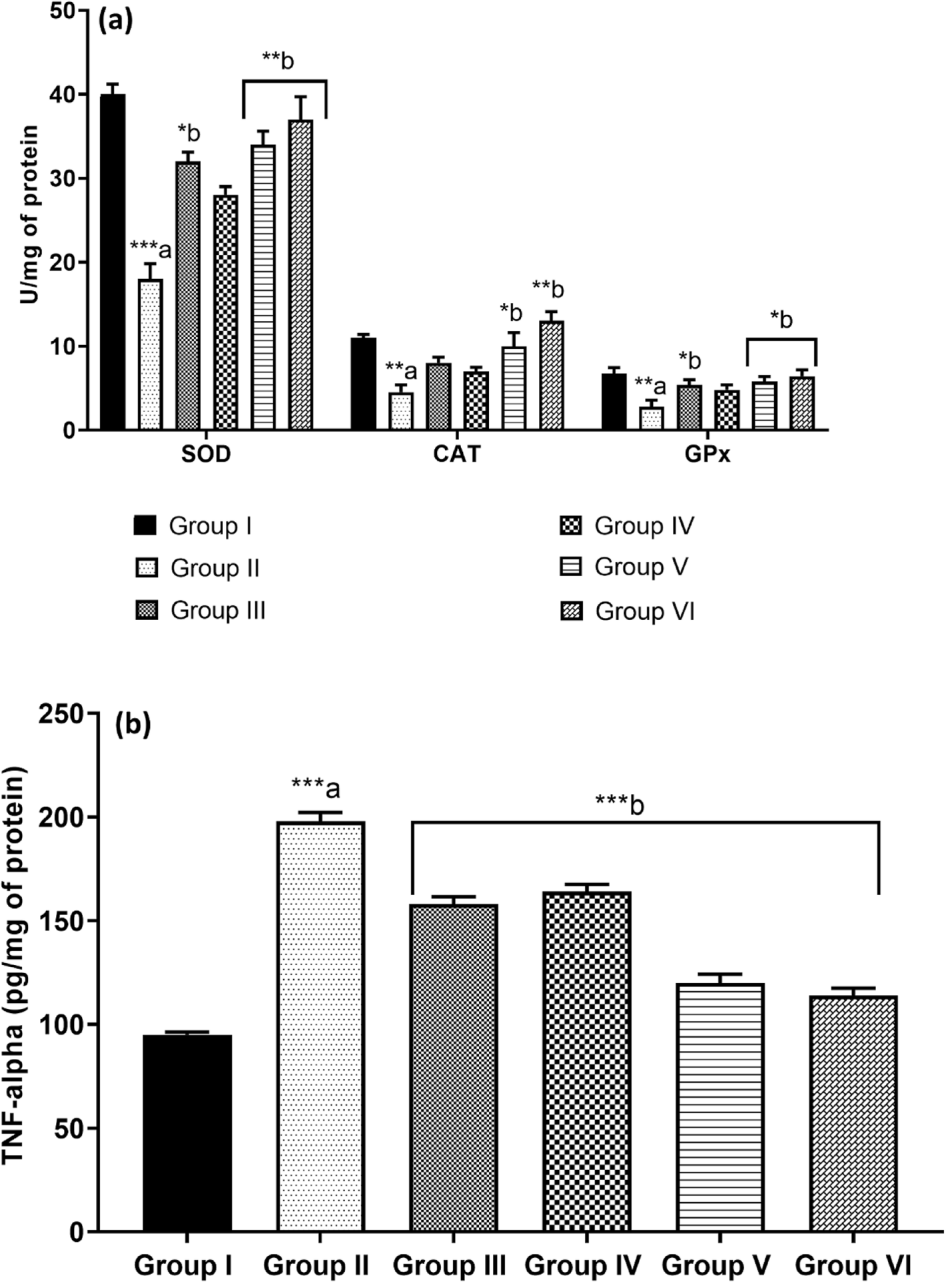
(**a**) Effect of kaempherol-3-rhamnoside and its combination on antioxidant enzymes was studied while ensuring originality. The statistical significance level was set at a *p*-value of 0.05, represented as *p < 0.05; **p < 0.01; ***p < 0.001 where “a” indicates normal control versus diabetic control and “b” represents diabetic control versus treatment groups; **(b)** The impact of kaempherol-3-rhamnoside and its combination on glucose levels and TNF-α was assessed in this study. Statistical significance was determined using a *p*-value of 0.05, whereby ***p < 0.001 indicated a significant difference between the normal control group and diabetic control group, while b-diabetic control versus treatment groups also exhibited statistical significance.

The results indicated a significant decrease in the levels of serum GSH, SOD, and CAT in the diabetic control group compared to the normoglycemic controls (*p* < 0.01). Conversely, treatment with kaempferol-3-rhamnoside, either alone or in conjunction with insulin, significantly elevated the serum levels of these antioxidants (*p* < 0.01). Notably, administration of kaempferol-3-rhamnoside at a dosage of 2.5 mg/kg did not enhance antioxidant levels when compared to diabetic controls. In contrast, the combination of kaempferol-3-rhamnoside with insulin elicited the most substantial increase in GSH, SOD, and CAT levels (*p* < 0.01), as depicted in Fig. 5a. These findings underscore the potential efficacy of this therapeutic approach in improving antioxidant status in individuals with diabetes, highlighting the importance of combination therapies in optimizing the therapeutic outcomes of such treatments.

### TNF-alpha level

In comparison with non-diabetic control mice, mice with diabetes demonstrated elevated levels of tumor necrosis factor-alpha (TNF-α) in the pancreas. Administration of kaempferol-3-rhamnoside at doses of 2.5 mg/kg or 5 mg/kg orally over a period of 4 weeks resulted in a reduction of pancreatic TNF-α levels. This effect was particularly pronounced when kaempferol-3-rhamnoside was co-administered with insulin therapy in diabetic mice, as illustrated in Fig. 5b.

### AMPK expression in liver

**Figure 6:**
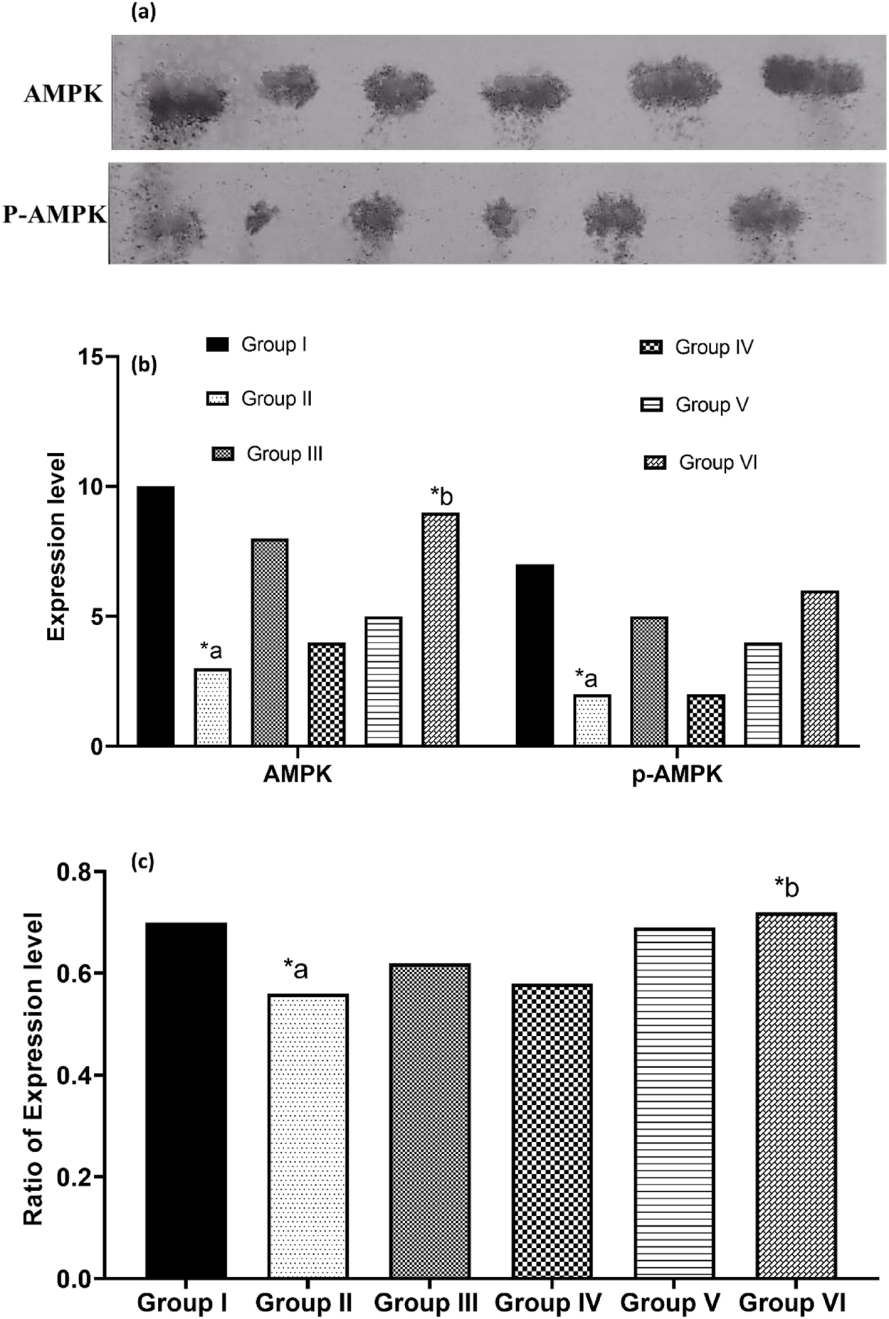
(**a**) The expressions of AMPK and *p*-AMPK in the liver were analyzed using Western blot; (**b**) kaempherol-3-rhamnoside and its combination’s impact on AMPK and *p*-AMPK was examined; **(c)** The ratio of pAMPK/AMPK expressions was calculated. A significance level of < 0.05 was deemed statistically significant, represented as *p < 0.05; where a denotes normal control versus diabetic control, while b represents diabetic control versus treatment groups.

In Fig. 6, a comprehensive examination of AMPK and its phosphorylated form (p-AMPK) in liver tissue via Western blotting reveals a notable decrease in both p-AMPK and total AMPK protein expression levels in diabetic mice compared to normal controls (*p* < 0.01). However, following treatment with kaempferol-3-rhamnoside combined with insulin, these levels exhibited a significant increase relative to the diabetic control group (*p* < 0.05). These results indicate that diabetes substantially reduces AMPK activity in the liver, but the administration of kaempferol-3-rhamnoside and insulin substantially enhances the p-AMPK/AMPK ratio, thereby activating AMPK function in hepatic tissues.

## Discussion

The animal model for diabetes used in this study involves administering STZ via a single injection of i.p., resulting in impaired insulin secretion even at high levels of glycemia and a moderate level of insulin resistance. This mixed model disease shows characteristics similar to both Type I and Type II diabetes mellitus^47^. The fact that kaempherol-3-rhamnoside can promote hypoglycemic effects in diabetic models indicates its ability to bypass insulin resistance and lower glycemia. This advantageous property allows for the reduction of glycemia even when insulin resistance is present. However, it is unlikely that kaempherol-3-rhamnoside improves insulin responsiveness, since no decrease in insulin levels was observed alongside unchanged glycemia. These findings demonstrate that there are no noticeable effects on the insulin levels of the control and STZ groups due to the consumption of kaempherol-3-rhamnoside.

Kaempherol-3-rhamnoside, a flavonoid, has shown the ability to lower blood sugar levels when given orally to STZ-induced diabetic mice. The authors suggested that kaempherol-3-rhamnoside triggers insulin signaling pathways that lead to increased glucose absorption by tissues outside the pancreas^48^. To support this idea, we refer to the research conducted by Tzeng et al. Their study revealed that kaempherol-3-rhamnoside activates traditional insulin signaling pathways in 3T3-L1 cells. This is achieved through the phosphorylation of key proteins such as the insulin receptor substrate 1, the insulin receptor itself, and other regulatory molecules within the cell. As a result, there is an increase in GLUT-4 translocation, a glucose transporter located on cell membranes that facilitates glucose uptake into tissues sensitive to insulin^49,50^.

The occurrence of oxidative stress is associated with various diseases, such as diabetes and its related complications. The production of free radicals or mitochondrial superoxide leads to the development of this condition, which can be caused by different mechanisms including elevated glycolysis and polyol pathway activation, non-enzymatic protein glycation, auto-oxidation due to excessive glucose levels in tissues, and decreased antioxidant enzyme levels^51^. Our study showed that in diabetic mice induced by alloxan, there was a significant reduction in the levels of antioxidant enzymes. This depletion may be related to oxidative stress-related harm experienced by both the serum and liver. The free radical scavenging system comprises both enzymes (SOD, CAT, and GPx) and non-enzymes (GSH, vitamin C, vitamin E) antioxidants that are tightly controlled in normal circumstances^52^. Antioxidants decreased and higher levels of TBARS were observed with diabetes^53^. After kaempherol-3-rhamnoside administration, there was an improvement in enzymatic antioxidant potential, while non-enzymatic oxidative stress markers reduced tissue damage caused by oxidative stress. Furthermore, free radical oxidation due to oxidative stress causes the formation of lipid peroxide in the membranes that leads to membrane dysfunction that leads to membrane dysfunction^54^. The use of kaempherol-3-rhamnoside has shown significant potential in the treatment of both microvascular and macrovascular diabetic complications. This is evidenced by the reduction in accumulated peroxides after administration, indicating a persuasive improvement in overall health outcomes.

To this, how kaempherol-3-rhamnoside lowers blood sugar, we examined the activity of PFK in key tissues that regulate glycemia—specifically liver tissue from healthy mice and those with STZ-induced diabetes. The primary pathway for cellular glucose consumption is glycolysis, which is highly dependent on PFK as a rate-limiting enzyme^50,55^. The effectiveness of kaempherol-3-rhamnoside in reversing impaired enzymatic activity and increasing PFK activity in liver tissue from diabetic mice is indicative of its ability to promote glucose utilization. Although the stimulation of glycolysis may be one possible mechanism for its hypoglycemic effects, it should be noted that other mechanisms are likely involved to ensure a sustained reduction in glycemia. In general, this evidence strongly supports the persuasive argument for the use of kaempherol-3-rhamnoside as an effective treatment option for diabetes^56^. According to this study, kaempherol-3-rhamnoside has been found to stimulate not just PFK but also other crucial glycolytic enzymes such as hexokinase (HK) and pyruvate kinase (PK). These findings suggest that the compound can enhance glucose utilization by cells, thus improving both catabolic and anabolic pathways along with overall energy balance. Interestingly, while intracellular ATP levels were observed to increase at all concentrations used, only kaempherol-3-rhamnoside was able to augment glucose catabolism (such as glucose consumption, lactate production, and metabolizing enzymes). It should be noted that insulin also exhibits a similar trend in which a higher concentration of hormones of hormones negates its stimulatory effects on metabolism^57,58^.

To our knowledge, only one investigation has documented the activating impact of kaempherol-3-rhamnoside on PFK. However, we could not locate any research linking flavonoids to improving phosphofructokinase (PFK) function and diabetes development^59^. Several flavonoids possessing hypoglycemic properties have been found to exhibit efficacy in various glucose-metabolizing enzymes. For example, rutin and quercetin, which are glycosylated flavonols, demonstrated the ability to regulate HK activity, as well as fructose 1,6-bisphosphatase and glucose 6-phosphatase^50,60^. Similarly, fisetin, another glycosylated flavanols, has shown potential in modulating PK, LDH G6PDH glycogen synthase and glycogen phosphorylase alongside the aforementioned enzymes^61^. Numerous studies have supported the effectiveness of flavanones, such as naringenin and diosmin, in the regulation of essential enzymes involved in carbohydrate metabolism, leading to reduced glucose levels^62,63^. This emphasizes that the control of these key metabolic pathways is a recurring process attributed to the glucose-reducing properties of flavonoids. It is crucial to address the pressing issue of developing improved treatments to manage glucose homeostasis and increase insulin sensitivity. AMP-activated protein kinase (AMPK) emerges as a prime contender, being a well-preserved serine/threonine kinase that triggers favourable effects on insulin responsiveness. Therefore, it stands out as an ideal therapeutic focus to effectively tackle Type 2 Diabetes (TD2).

AMPK, an enzyme that monitors cellular energy levels, became active when depleted. It then sent signals to increase glucose absorption in skeletal muscles and promoted fatty acid oxidation in adipose tissue (as well as other tissues). Additionally, it curbed hepatic glucose production^64^. The evidence supporting AMPK dysregulation in both animals and humans with metabolic syndrome or type II diabetes is quite compelling. In addition, activating AMPK through physiological or pharmacological interventions has been found to lead to improvements in insulin sensitivity and overall metabolic health^65^. There is an abundance of pharmaceutical drugs, natural substances, and hormones that have the ability to activate AMPK through direct or indirect means. Some examples of these compounds include metformin and thiazolidinediones, which are already being used for the treatment of Type II diabetes^66^. This research also demonstrated that kaempherol-3-rhamnoside plays a role in activating AMPK, which then controls metabolic enzymes.

Kaempherol-3-rhamnoside can activate AMPK by increasing the cellular AMP: ATP ratio or by modulating the activity of upstream kinases. One of the primary pathways involved in Kaempherol-3-rhamnoside-mediated AMPK activation is through activation of the tumour suppressor kinase LKB1. kaempherol-3-rhamnoside can promote the phosphorylation and activation of LKB1, which in turn phosphorylates and activates AMPK. kaempherol-3-rhamnoside has also been reported to activate AMPK through the CaMKKβ pathway. kaempherol-3-rhamnoside can stimulate CaMKKβ, which phosphorylates and activates AMPK independently of changes in the AMP: ATP ratio.

Kaempherol-3-rhamnoside may also directly bind to AMPK and influence its activation. Studies have suggested that kaempherol-3-rhamnoside can bind to AMPK's γ subunit and promote conformational changes that enhance AMPK activation. Activation of AMPK by kaempherol-3-rhamnoside leads to various downstream effects that contribute to its potential therapeutic benefits. AMPK activation can increase glucose uptake, enhance fatty acid oxidation, inhibit gluconeogenesis (glucose production), promote mitochondrial biogenesis, and regulate gene expression involved in energy metabolism. It is important to note that the exact molecular mechanisms underlying kaempherol-3-rhamnoside activation of AMPK and p-AMPK may vary depending on the cell type, experimental conditions, and concentrations used. Further research is needed to elucidate the detailed mechanisms and signaling pathways involved in kaempherol-3-rhamnoside effects on AMPK activation. Additionally, it is worth mentioning that the activation of AMPK by kaempherol-3-rhamnoside is just one aspect of its overall biological activity, and its effects on other cellular processes and pathways may also contribute to its potential therapeutic effects.

## Conclusions

The findings of this research suggest that kaempherol-3-rhamnoside could possess potential antidiabetic properties independently or in combination with insulin. The objective of the study was to assess how kaempherol-3-rhamnoside affected the AMPK pathway, metabolic enzymes, glycolytic enzymes, gluconeogenic enzymes, and NADP-linked lipogenic enzymes in liver tissues obtained from mice who had developed diabetes after STZ induction.

Upon analysis, the results showed that in diabetic mice, there was a decrease in glycolytic enzyme activity while gluconeogenic enzyme activities increased. This suggests an impaired glucose metabolism. In particular, these mice also exhibited reduced lipid-based enzyme activity. Interestingly, normalising the enzymatic activities associated with both glucose and lipid metabolism through treatment with kaempherol-3-rhamnoside led to therapeutic benefits. As such, it can be concluded that kaempherol-3-rhamnoside is effective against dysfunctional glucose metabolism found among diabetic subjects.

In addition, it was revealed that kaempherol-3-rhamnoside treatment triggered the activation of AMPK activity in liver tissues among diabetic mice. An increase in the p-AMPK/AMPK ratio indicated that this activation is vital. There had been a prior suppression of AMP kinase activity in these mice before its successful recovery by kaempherol-3-rhamnoside treatment. These findings point to the prospective role of kaempherol-3-rhamnoside as a regulator of metabolic processes related to diabetes by simplifying modulation throughout the AMPK pathway. Compelling evidence supporting the efficacy of kaempherol-3-rhamnoside in managing diabetes was obtained from a biochemical investigation, which demonstrated its ability to rejuvenate cellular activity on a molecular scale.

Overall, this study contributes to our understanding of the mechanisms underlying kaempherol-3-rhamnoside anti-diabetic effects and sheds light on its role in regulating glucose and lipid metabolism, as well as the AMPK pathway. To examine the complete therapeutic potential of kaempherol-3-rhamnoside in in the treatment of diabetes and its complications, additional research and clinical studies are required.

## Data Availability

All data used for this study are contained in this article.
